# Crystallinity of Amphiphilic PE-b-PEG Copolymers

**DOI:** 10.3390/polym14173639

**Published:** 2022-09-02

**Authors:** Sophie Bistac, Maurice Brogly, Diane Bindel

**Affiliations:** Laboratoire de Photochimie et d’Ingénierie Macromoléculaires, Université de Haute Alsace, 3b rue Alfred Werner, 68093 Mulhouse, France

**Keywords:** PE-b-PEG copolymers, optical microscopy, differential scanning calorimetry, PE, PEG, crystallization

## Abstract

The crystallinity and the growth rate of crystalline structures of polyethylene glycol and polyethylene blocks in polyethylene-b-polyethylene glycol diblock copolymers (PE-b-PEG) were evaluated and compared to polyethylene and polyethylene glycol homopolymers. Melting and crystallization behaviours of PE-b-PEG copolymers with different molecular weights and compositions are investigated by differential scanning calorimetry (DSC). The polyethylene/polyethylene glycol block ratio of the copolymers varies from 17/83 to 77/23 (weight/weight). The influence of the composition of PE-b-PEG copolymer on the ability of each block to crystallize has been determined. Thermal transition data are correlated with optical polarized microscopy, used to investigate the morphology and growth rate of crystals. The results show that the crystallization of the polyethylene block is closer to the polyethylene homopolymer when the copolymer contains more than 50 wt. % of polyethylene in the copolymer. For PE-b-PEG copolymers containing more than 50 wt. % of polyethylene glycol, the polyethylene glycol block morphology is almost similar to the PEG homopolymer. An important hindrance of each block on the crystallization growth rate of the other block has been revealed.

## 1. Introduction

Amphiphilic diblock copolymers are macromolecules with a hydrophobic block and a hydrophilic block. These copolymers are able to self-organize in bulk or in solution. These specific properties have attracted a great deal of research interest in the past decade due to their potential in applications as industrial surfactants [1]. The major types of block copolymers, such as those made from ethylene oxide (EO) and propylene oxide (PO) constitute a vast majority of diblock copolymer on the market [2]. As an example, the amphiphilic properties of EO-PO block copolymers [3] allow their use as surfactants, emulsifiers, emulsion stabilizer [4], foam stabilizers [4], drug carriers [5] or detergents [4].

Crystallization of diblock copolymers composed of one amorphous block and one semi-crystalline block has been studied in the literature [6,7] as PEO-b-PB [8,9], PEO-b-PS [10] or PS-b-PP [11]. The synthesis [12] and the melting behaviour [13] of crystalline–crystalline diblock copolymers are presented in the literature, but no specific study is dedicated to the bulk crystallization of amphiphilic diblock copolymers containing two semi-crystalline blocks with different proportions. This work is focused on the study of amphiphilic block copolymers containing two semi-crystalline blocks, both able to crystallize.

Commonly used as surfactants, polyethylene-block-polyethylene glycol copolymers (PE-b-PEG) are amphiphilic, and both blocks, hydrophilic PEG and hydrophobic PE, might be expected to crystallize into two distinctive crystalline phases.

The objective of this work is to study the effect of the block ratio on the PE-b-PEG crystallinity. The effects of the molecular weight and the weight ratio of the two polymer blocks on the PE-b-PEG crystallinity will be evidenced by the characterization of various PE-b-PEG copolymers of different compositions. The thermal properties and specifically the crystallinity degree of each block will be characterized by differential scanning calorimetry. The morphology and the growth rate of the crystalline phase of each block are investigated by optical microscopy under polarized light in combination with a hot-stage. In order to evidence the influence of the blocks ratio, the results obtained for PE-b-PEG copolymers are compared with the thermal and morphological properties of the PE and PEG homopolymers.

## 2. Materials and Methods

### 2.1. Materials

**Figure 1:**
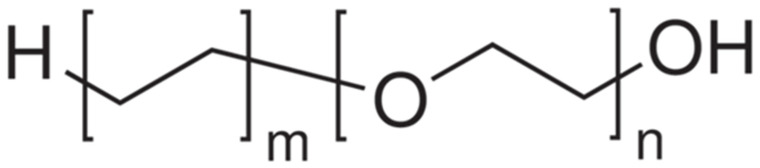
Formula of the PE-b-PEG diblock copolymer.

Different PE-b-PEG copolymers have been used. The general formula of these copolymers is given in Figure 1.

PE-b-PEG copolymers of various molecular weights were supplied by Sigma Aldrich (Saint-Quentin-Fallavier, France). PE-b-PEG copolymers are conditioned as pellets. Two homopolymers were also studied: polyethylene glycol (PEG) conditioned as flakes (Sigma Aldrich, *M_n_* = 2050 g·mol^−1^), and polyethylene (PE) conditioned as powder (Sigma Aldrich, *M_n_* = 1700 g·mol^−1^). These PE and PEG homopolymers have low molecular weights, closed to the molecular weights of PE blocks and PEG blocks in the studied copolymers. However, molecular weights of PE and PEG blocks of the copolymers are smaller than those of the corresponding homopolymers. Shorter chains lengths are not provided by the supplier, homopolymers with the closest chains lengths have been thus chosen for the study. The following results obtained can be influenced by the non-equal chain lengths between copolymer blocks and the homopolymers, but the main interest of this study is to compare diblock copolymers. The results will be consequently discussed by referring to the properties of PE and PEG blocks of close molecular weight to the blocks of the copolymer existing in the literature.

The ethylene/ethylene oxide ratio and the number average molecular weight of the copolymers indicated by the supplier have been verified by proton nuclear magnetic resonance (^1^H NMR). The average values of these data are presented in Table 1.

### 2.2. Characterization

#### DSC and Optical Microscopy Analysis

Thermal transitions and associated temperatures are determined by DSC measurements. A DSC Q200 from TA Instruments (Guyancourt, France) was used. Samples of ca. 10 mg were placed in sealed aluminum pans; an empty aluminum pan was used as a reference. Two temperature ramps were applied in order to cancel the thermal history. A heating rate of 10 °C/min and a cooling rate of 10 °C/min were applied between −80 °C and 160 °C.

The morphology of PE, PEG and PE-b-PEG copolymers was observed by the use of an Olympus BX51 (Rungis, France) optical microscope equipped with polarized light. The polarized light is particularly interesting to differentiate the crystalline and the amorphous phases. Magnifications of ×500 and ×100 were used. The microscope is also equipped with a CCD camera connected to a computer in order to record each step of the melting and the crystallization. The temperature of the sample was controlled by the use of a Linkam LTS350 hot-stage coupled to the microscope.

In order to be observed by optical microscopy in transmission mode, thick films of PE, PEG and PE-b-PEG were obtained by casting a polymer layer between two glass slides. A glass/pellet/glass sandwich is placed in oven and heated up to a molten state at 120 °C, and kept at this temperature during 15 min. This temperature is above the melting point of PE and PEG, ensuring a complete flowing of both blocks. After 15 min in the oven, the glass slides were separated in such a way that about 200 µm of melted polymer was deposited on each of them. Both glass slides were cooled at ambient temperature.

The melting of polymers was recorded during a heating ramp from the ambient temperature to 120 °C at a rate of 10 °C/min. The crystallization of polymers has been recorded during a cooling ramp running from 120 °C to the ambient temperature (20 °C) at a rate of 2 °C/min.

The spherulites and lamellas sizes were directly measured on the images recorded by the CCD camera. The initial and final crystallization temperatures were determined from the video recording the crystallization. The growth rate of these crystalline structures was obtained from the evolution of the size as a function of time, directly determined from the videos recorded by the CCD camera.

## 3. Results

### 3.1. PEG Homopolymer

#### 3.1.1. Thermal Analysis

**Figure 2:**
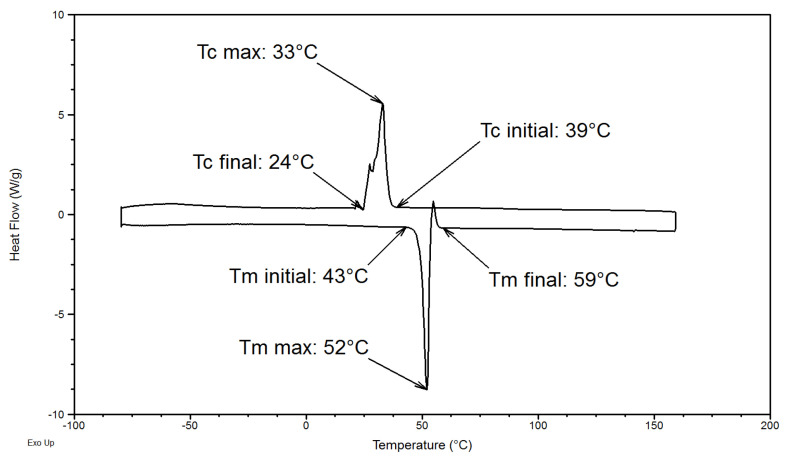
Differential Scanning Calorimetry DSC curves of PEG homopolymer during the heating (down) and the cooling (up) ramps.

Figure 2 shows DSC curves of semi-crystalline PEG homopolymer and its characteristic thermal transitions. The crystalline phase melting occurs during the heating ramp between 43 °C and 59 °C. The maximal melting temperature T_m max_ (maximum of the endothermic peak) is equal to 52 °C. The crystallization occurs during the cooling ramp between 39 °C and 24 °C. The maximal crystallization temperature T_c max_ (maximum of the exothermic peak) is equal to 33 °C.

The degree of crystallinity *X_c_* was calculated using the formula below, where Δ*H_m_*^0^ is the melting enthalpy of 100% crystalline PEG (196.8 J/g [14,15]), and Δ*H_m_* is the melting enthalpy of PEG under investigation (195.7 J/g).

$$X_{c}=\frac{\Delta H_{m}}{\Delta H_{m}^{0}}$$

Very close heat of fusion of 191 J/g (*M_n_* = 8000 g·mol^−1^) [15] and 183 J/g (*M_n_* = 3400 g·mol^−1^) [14] have been found in the literature for PEG with similar chains lengths indicating the high crystallinity of PEG homopolymers. The degree of crystallinity of this PEG homopolymer is equal to 95% and is very close to the degree of crystallinity of PEG homopolymers of different molecular weights studied and described in the literature. Several studies reveal that the degree of crystallinity increases as the molecular weight of the homopolymer does: for PEG homopolymers varying from *M_n_* = 1000 g·mol^−1^ to *M_n_* = 20,000 g·mol^−1^, the degree of crystallinity varies from 85% to 99% [14,16,17]. The tendency and the order of magnitude are confirmed between experiments and the literature; the melting enthalpy and the crystallinity degree increases as the molecular weight of the polymer increases. The differences observed between the experimental melting enthalpy and crystallinity degree obtained and the values extracted from literature can be attributed to the initial and final melting temperatures used for the peak integration. Due to the very high crystallinity of PEG, the sensitivity of the DSC does not allow determination of the glass transition temperature (Tg) of PEG, which is commonly observed between −60 °C and −70 °C in the literature [18,19].

#### 3.1.2. Morphology and Growth Rate of PEG Homopolymer Crystalline Structures

**Figure 3:**
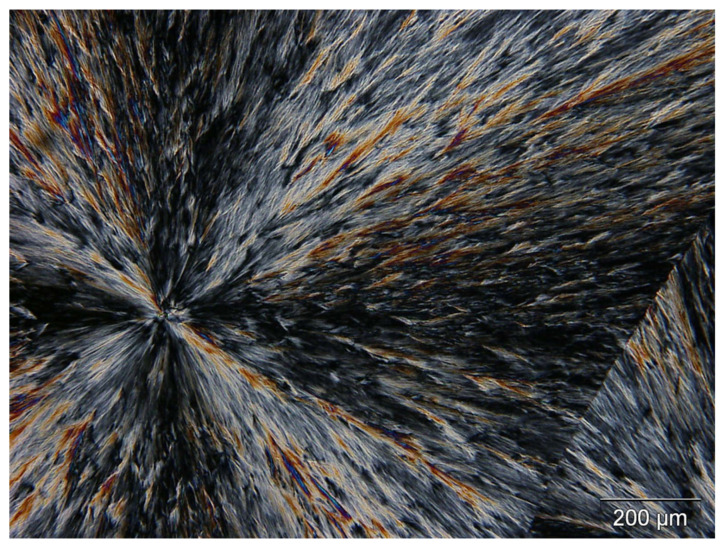
PEG spherulites observed by optical microscopy in combination with polarized light at ambient temperature.

The morphology of bulk PEG was observed by optical microscopy under polarized light at ambient temperature. PEG film is obtained by deposition at 120 °C (melted state) on a glass slide and is cooled rapidly at ambient air (see Figure 3). Large spherulites varying from 200 µm to 2 mm diameter were observed. These structures are characteristic of the crystalline phase of polyethylene glycol [16,20]. The PEG spherulites completely cover the glass slide confirming the high crystallinity of the PEG homopolymer.

Melting and crystallization temperatures of PEG have been also determined by optical microscopy thanks to the coupled CCD camera recording the spherulites melting and crystallization during the heating and the cooling ramps, respectively (see Table 2). The initial melting temperature T_m initial_ and the final melting temperature T_m final_ correspond to the first disappearance of spherulites and to the complete disappearance of spherulites, respectively. The initial crystallization temperature T_c initial_ and the final crystallization temperature T_c final_ correspond to the first appearance of spherulites and to the complete covering of the glass slide with PEG spherulites, respectively.

The melting and crystallization temperatures of PEG observed by optical microscopy are very close to those observed by DSC (see Table 2). Some differences between DSC and optical microscopy temperatures could be explained by the different heating and cooling rates applied in both methods. In order to reveal some difference due to the heating or cooling ramp in thermal properties, DSC curves have been performed with a heating rate of 10 °C/min and a cooling rate of 2 °C/min as applied to the heating stage coupled with the microscope.

The characteristic temperatures observed by DSC were not influenced by the different rates applied. An explanation can be given to the more important difference between crystallization temperatures. As the heating stage is cooled by ambient air during crystallization, the cooling step at 2 °C/min induces a less precise control of the temperature below 35 °C. Moreover, if primary crystallization is easily visible on optical images, the secondary crystallization is less visible, and complete covering of the image by spherulites does not mean that secondary crystallization is ended. The secondary crystallization is particularly visible in Figure 2 where the crystallization peak of PEG is asymmetric. This asymmetry can be attributed to the secondary crystallization occurring after the primary crystallization. This secondary crystallization can be associated to a phenomenon of primary crystalline phase improvement, or to the crystallization of the amorphous phase rejected from the primary crystallization and trapped between or inside the spherulites [21].

**Figure 4:**
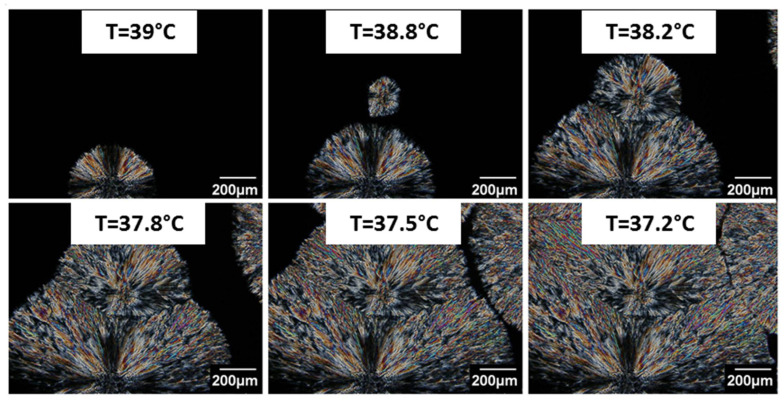
PEG spherulite crystallization observed by optical microscopy in combination with polarized light during a cooling ramp.

The melting temperatures observed by DSC and optical microscopy are different even if the heating rates applied are the same for both techniques. Using optical microscopy, the area of interest is a circle of a few millimeters in diameter. The melting event might have thus begun for lower melting temperatures elsewhere. From Figure 4, obtained by optical microscopy coupled with a heating stage during a cooling ramp, it is possible to determine the size and the growth rate of PEG spherulites as a function of the crystallization temperature.

**Figure 5:**
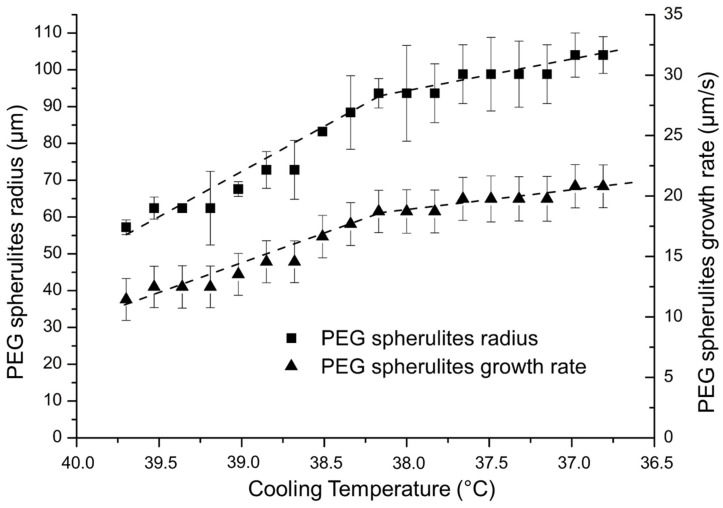
Evolution of PEG spherulite radius and growth rate as a function of the cooling temperature determined by optical microscopy.

The evolution of the spherulites size and growth rate as a function of the crystallization temperature is presented in Figure 5. The mean value is obtained from three reproducible measurements among five samples. The reproducibility from one sample to another is good and supported by the low standard deviation observed in Figure 5. The PEG spherulite size increases rapidly to 90 µm between 39.8 °C and 38.1 °C, and less rapidly between 38.1 °C and 36.8 °C. The growth rate exhibits the same tendency. Indeed, as the crystallization temperature decreases, the melted polymer tends to be more viscous and thus limits the growth and the size of the PEG spherulites.

The average growth rate of spherulites in PEG homopolymer is equal to 17 µm/s. This represents a reference to reveal the influence of the PE block in PE-b-PEG diblock copolymer. It is also important to point out that the growth rate decreases as the crystallization temperature is increased. The spherulitic morphology of crystallized PEG has been observed in many studies [14,16,17], revealing spherulites growing up to 4 cm diameter and reaching a rate of 0.14 cm/s from 56 °C to 50 °C [14,20] for PEG with Mw = 200,000 g·mol^−1^. The value of 17 µm/s is very slow compared to 0.14 cm/s, but might be explained by the different cooling ramp used in the quoted reference, and also by the different conditioning of PEG 2,000,000 g·mol^−1^ as powder.

### 3.2. PE Homopolymer

#### 3.2.1. Thermal Analysis

**Figure 6:**
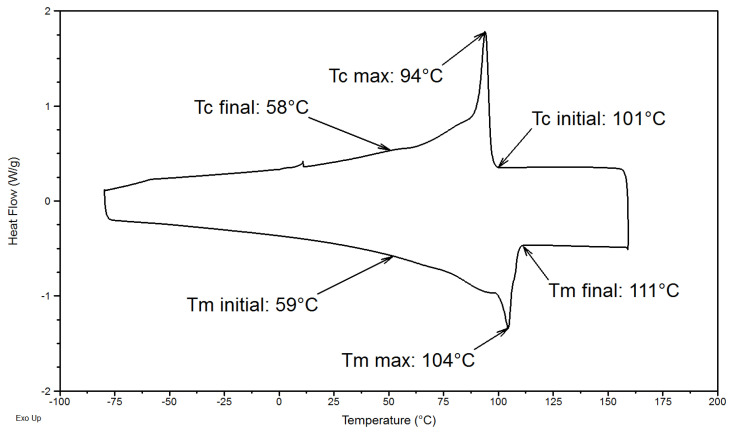
DSC curve of PE homopolymer during the heating (down) and the cooling (up) ramps.

Bulk thermal properties of PE homopolymer were also investigated. Figure 6 shows DSC curves of the PE sample and its characteristic thermal transitions. The melting occurs during a heating ramp between 59 °C and 111 °C. The crystallization of the crystalline phase occurs during a cooling ramp between 101 °C and 58 °C. The curves did not allow determination of the glass transition of PE.

Melting and crystallization peaks are very wide, and the limits for peaks integration are therefore difficult to define. The degree of crystallinity *X_c_* of PE homopolymer was calculated using Δ*H_m_*^0^, the melting enthalpy of 100% crystalline PE (285 J/g [22]) and Δ*H_m_,* the melting enthalpy of the studied PE (126 J/g). The degree of crystallinity of this PE is equal to 44%.

The very wide melting peak indicates that the crystalline organisation is less perfect compared with PE with a higher molecular weight. Peacock [23] and Mandelkern [24] have discussed the influence of the molecular weight on the melting enthalpy and the degree of crystallinity of various PE. The narrowest melting ranges are exhibited for low molecular weight PE [23]. The melting temperatures decrease as the molecular weight increases [23]. From a low density polyethylene to a high density polyethylene, the melting enthalpy varies between 88 J/g (*X_c_* = 30%) and 222 J/g (*X_c_* = 77%), respectively [23]. Considering the low molecular mass (*M_n_* = 1700 g·mol^−1^) and the crystallinity degree (44%) related to the melting enthalpy (126 J·g^−1^), the experimental PE homopolymer studied belongs to the low density polymers, which is useful in the comprehension of the PE block in the PE-b-PEG copolymer.

#### 3.2.2. Morphology and Growth Rate of PE Crystalline Structures

**Figure 7:**
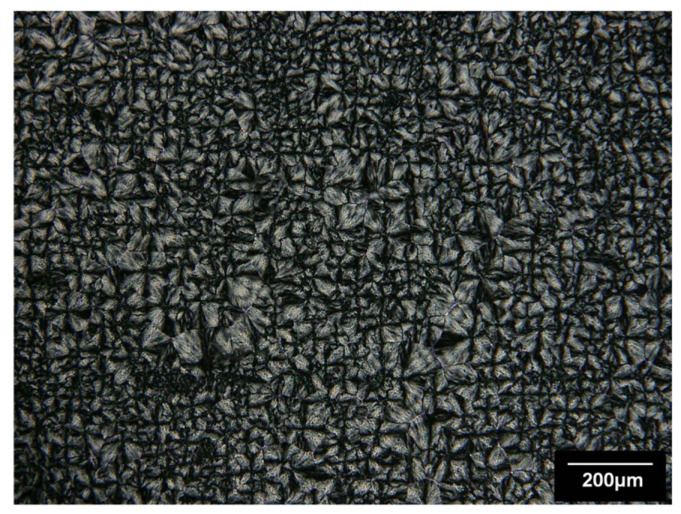
PE spherulites observed by optical microscopy in combination with polarized light at ambient temperature.

The morphology of polyethylene was observed by optical microscopy under polarized light at ambient temperature after being deposited at the melting state (120 °C) on a glass slide and rapidly cooled at ambient temperature (Figure 7). Spherulites varying from 20 µm to 100 µm diameter were observed. By using polarized light, the amorphous zones are black and the crystalline zones are grey. The PE spherulites completely cover the glass slide, and one can notice that spherulites are composed of dark zones alternating with light grey zones. Indeed, PE spherulites are composed of crystalline lamellas alternating with amorphous zones giving characteristic “Malta crosses” under crossed polarization.

**Figure 8:**
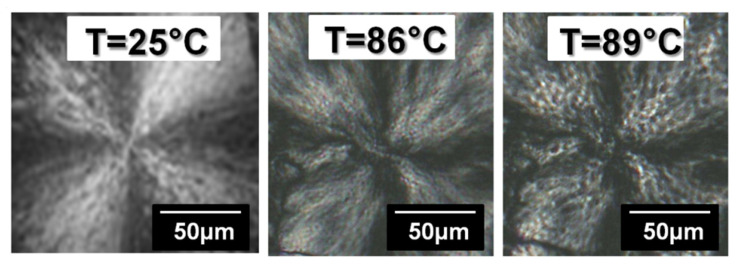
Observation by optical microscopy with a polarized light of PE spherulites melting behaviour.

The melting of PE was recorded between 30 °C and 110 °C by optical microscopy equipped with a CCD camera and coupled with a hot-stage applying a heating ramp from the ambient temperature to 120 °C at 10 °C/min. Between 86 °C and 89 °C (see Figure 8), one can notice some morphological differences of the spherulites. Indeed, crystalline structures formed during the secondary crystallization melt.

Melting and crystallization temperatures of bulk PE were determined by optical microscopy during a cooling and a heating ramp applied to a hot-stage. These temperatures were compared to the characteristic temperatures obtained by DSC and gathered in Table 3.

The melting and the crystallization temperatures of pure PE determined by optical microscopy are very close to the one observed on the DSC thermogram, except for T_c final_. When PE crystallizes, spherulite growth until 50 °C is observed. Below 50 °C, any changes were seen as secondary crystallization and were less observable by optical microscopy.

**Figure 9:**
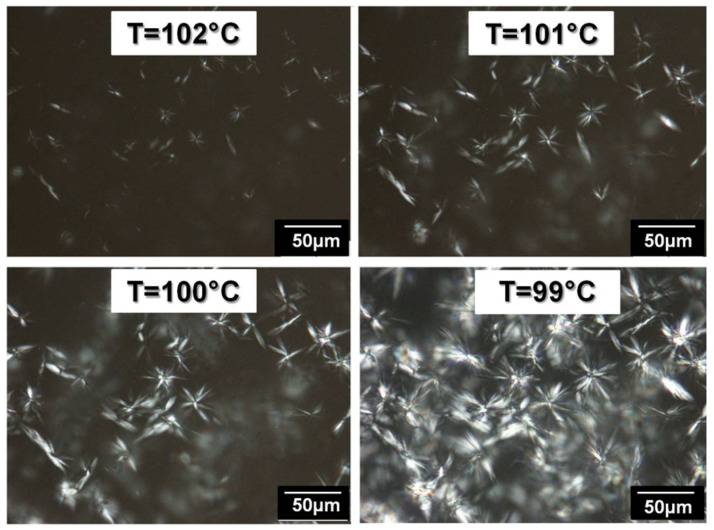
PE spherulites crystallization observed by optical microscopy with polarized light during a cooling ramp.

Figure 9 shows the crystallization of PE during a cooling ramp (120 °C→25 °C at 2 °C/min) applied to a hot-stage and coupled to the optical microscope. The nucleation of the crystalline spherulites in the amorphous phase is clearly visible in the crystallization temperature range.

**Figure 10:**
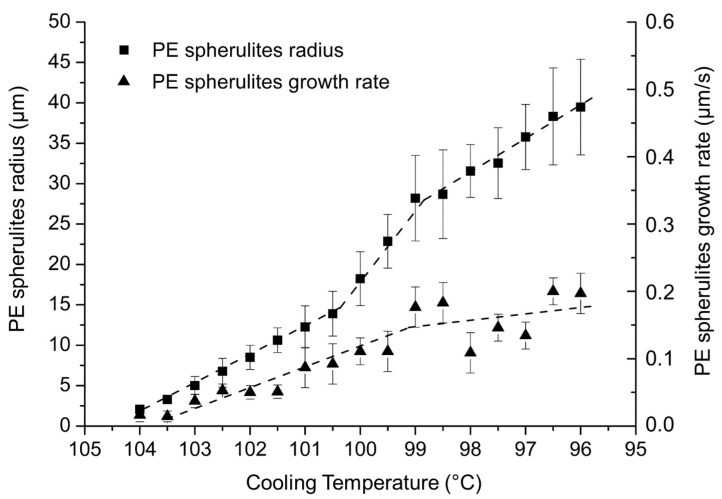
Evolution of the PE spherulite radius and growth rate as a function of the cooling temperature determined by optical microscopy.

The evolution of the PE spherulite radii and the spherulites growth rates as a function of the PE crystallization temperature are presented in Figure 10. The mean value is obtained from three reproducible measurements among five samples. The reproducibility from one sample to another is good and supported by the low standard deviation observed in Figure 10. Spherulite radii increase rapidly until 14 µm between 104 °C and 96 °C, revealing the high mobility of short PE chains. They reach their maximal radius of 40 µm at 95.5 °C.

The evolution of the PE spherulite growth rate as a function of the crystallization temperature is also presented in Figure 10. The spherulitic growth rate increases rapidly between 104 °C and 99 °C, up to 0.5 µm/s, and then slows from 99 °C to 95.5 °C.

Below 95 °C, spherulites are growing in a different plane from the one focused by the microscope and that growth cannot be recorded. The crystallization temperatures are very close to LDPE spherulitic crystallization observed between 117 °C [25] and 125 °C [26]. These crystallization temperatures mentioned in the literature are higher than the experimental crystallization temperatures of PE. Indeed, the crystallization temperature and growth rate quoted in the reference corresponds to the lowest molecular weight, equal to *M_n_* = 3600 g·mol^−1^ [26]. As the molecular weight of the PE homopolymer studied here is more than two-fold smaller, the crystallization temperature is thus lower than 125 °C. It is important to notice that the growth rate of spherulites increases as the cooling temperature decreases, and that a plateau is observed at 0.2 µm/s (taking into account the standard deviation) when PE spherulites reach their maximal size.

Two different morphologies of polyethylene crystalline structures are described in the literature: lamellas [22] or spherulites [27,28].

PE lamellas crystallizing at 2 µm/s were observed by Abo el Maaty [29]. PE spherulites crystallizing at rates ranged between 0.4 µm/s [25] and 100 µm/s [28] are mentioned in the literature. The low rate of spherulite crystallization of PE at 0.2 µm/s (see Figure 10) is in accordance with the low rate of spherulite crystallization of LDPE observed by Tavachai [25].

### 3.3. Crystallinity of PE-b-PEG Copolymers

Both PE and PEG homopolymers are semi-crystalline, and PE and PEG blocks are consequently able to crystallize in PE-b-PEG copolymers. PEG homopolymer presents a melting temperature lower than PE homopolymer and a very fine peak representative of a high crystalline organization. The aim of this part is to evidence the influence of each block on the crystallinity, the morphology and the growth rate of the crystalline structures of PE-b-PEG diblock copolymers.

#### 3.3.1. Thermal Analysis

**Figure 11:**
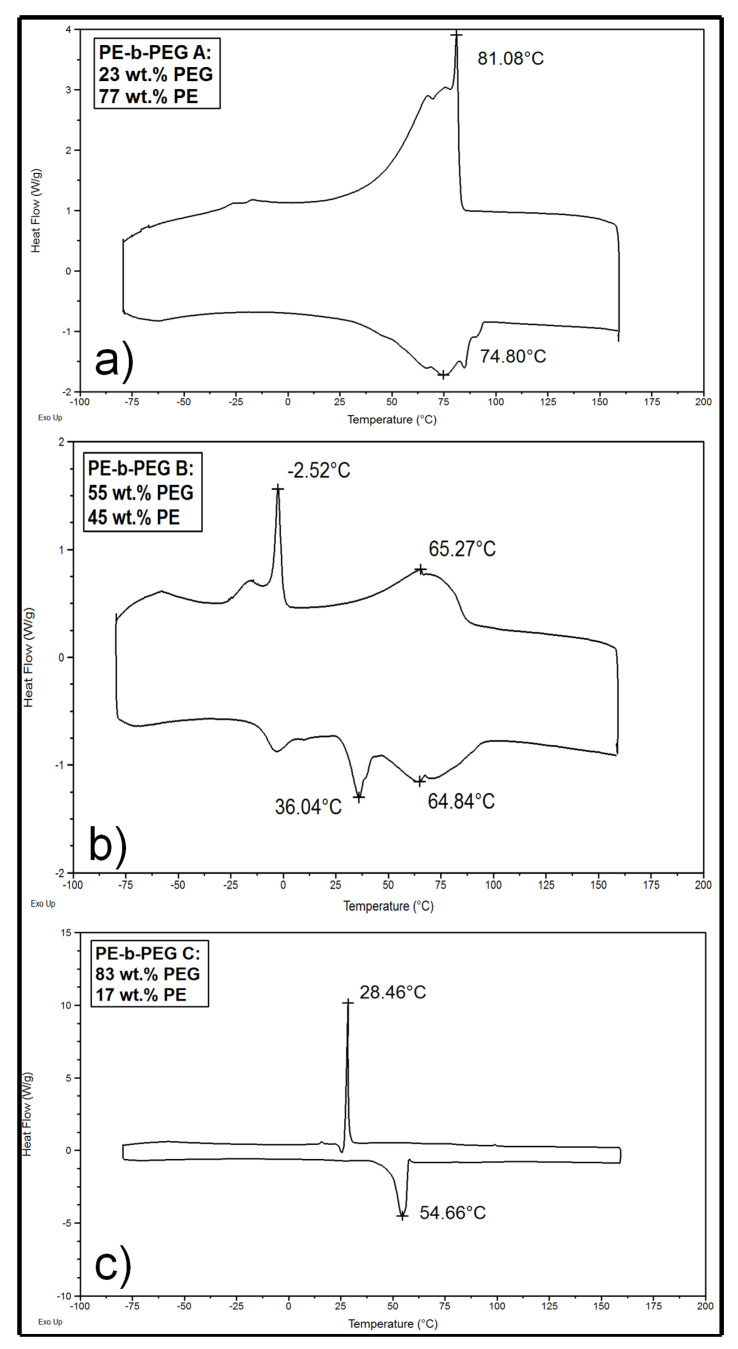
DSC curves of (**a**) PE-b-PEG A, (**b**) PE-b-PEG B and (**c**) PE-b-PEG C during the heating (down) and the cooling (up) ramps.

Figure 11 shows DSC heating and cooling thermograms of PE-b-PEG of different molecular weights and different blocks ratios. DSC curves obtained for PE-b-PEG A, PE-b-PEG B and PE-b-PEG C show one to two melting and crystallization peaks depending on the diblock composition. As mentioned for PE and PEG homopolymers, glass transitions are not detectable on the DSC curves of PE-b-PEG copolymers. In the following part, dealing with the crystallinity of PE-b-PEG copolymers, the study of diblock copolymers with highly different block ratios, PE-b-PEG C and PE-b-PEG A, are presented first, and then the study of a diblock with an intermediate composition, PE-b-PEG B, is presented (see Figure 11).

When the PE block is in the majority in the diblock, as is the case for PE-b-PEG A containing 77 wt. % PE, one very wide endothermic peak attributable to the PE crystalline phase melting is observed (Figure 11a). The melting enthalpy of this peak is equal to 275 J/g, corresponding to a degree of crystallinity equal to 96% for the PE blocks. Despite the high content of PE in PE-b-PEG A, differences in the shape and the temperature range of melting peaks are observed when compared to the PE peaks of PE homopolymer in Figure 6. The melting of the organized PE lamellas occurring at 104 °C in PE homopolymer (see Figure 6) is not observed in PE-b-PEG A. Indeed, the melting peak is shifted (T_m max_ = 74 °C) to a lower melting temperature, indicating that the PE crystals are not as well organized as in the PE homopolymer. The DSC curves obtained for PE-b-PEG A show a distribution of less organized crystalline structures with different melting and crystallization temperatures compared to the DSC curves of the PE homopolymer, relevant to the influence of the PEG blocks on the PE blocks crystallization. Moreover, no single contribution at 52 °C or lower temperatures, characteristic of the PEG homopolymer melting, is observed in Figure 11a, indicating that PEG melting is also highly perturbated by PE sequences. The short PE block length is able to explain the shift of the melting peak. Indeed, shorter PE chains crystals require less energy to be disorganized. The hypothesis of a PEG block hindrance on the thermal properties of the PE phase in PE-b-PEG A should also be considered.

The PE-b-PEG C copolymer containing a majority of PEG block in its composition was also analyzed by DSC in order to reveal the influence of the block ratio on the thermal properties and on the crystallinity of PE-b-PEG copolymers (Figure 11c). The DSC thermogram curves of this copolymer are very close to the one observed for the PEG homopolymer (see Figure 2) as the copolymer contains 83 wt. % PEG. Indeed, one single peak for crystallization and melting appears in the characteristic temperature range of the homopolymer PEG. The melting enthalpy of this single melting peak is equal to 162 J/g, corresponding to a crystallinity degree of 82% for PEG blocks. The influence of the PE block on the PEG crystallinity in PE-b-PEG C is revealed by the differences between the melting peak shape of the PEG homopolymer (Figure 2) and PE-b-PEG C (Figure 11c). The initial melting temperature of the PEG homopolymer is well defined at 43 °C, whereas the PEG initial melting temperature in PE-b-PEG C is slightly wider, between 35 °C and 45 °C. Even if no peaks in the area of the PE melting (or PE crystallization during cooling) zone can be seen on the DSC curve of PE-b-PEG C, the melting peak indicates that PE blocks do not crystallize but are able to influence the PEG block crystallization.

In order to more deeply understand the block ratio influence on PE-b-PEG copolymer crystallinity, a PE-b-PEG B (55 wt. % PEG) with an intermediate block composition was investigated by DSC and is presented in Figure 11b. Two distinct melting peaks and two distinct crystallization peaks can be observed. These peaks, corresponding to the melting of the two different blocks present in the copolymer, are wide and not well-defined. The melting temperatures of both blocks are shifted to the lower melting temperatures compared to the homopolymer melting temperatures: T_m max_ for PEG is equal to 36 °C and T_m max_ for PE is equal to 65 °C.

The same tendency is observed for the crystallization temperatures. The melting peak of the PEG blocks presents a melting enthalpy of 32 J/g corresponding to a crystallinity degree of 16% for the PEG blocks. The melting peak of the PE blocks presents a melting enthalpy of 77 J/g corresponding to a crystallinity degree of 27% for the PE blocks.

An important effect of one block on the ability of the other block to crystallize is evidenced. When a block is not strongly preponderant in a PE-b-PEG copolymer, each block is able to crystallize. Since the crystalline characteristics (T_m_, T_c_, *X_c_*) of each block are modified by the presence of the other block, the study of the crystalline morphologies can help us to understand the crystallinity behaviours of PE-b-PEG copolymers.

#### 3.3.2. Morphological Analysis and Crystalline Growth Rate Determination

##### PE-b-PEG C (83 wt. % PEG) Copolymer

The morphology of PE-b-PEG C containing 83 wt. % PEG was observed by optical microscopy under cross polarization. In order to obtain information about the crystalline structures of a copolymer containing a high proportion of PEG block, a heating and a cooling ramp were applied successively to the copolymer while a camera recorded.

**Figure 12:**
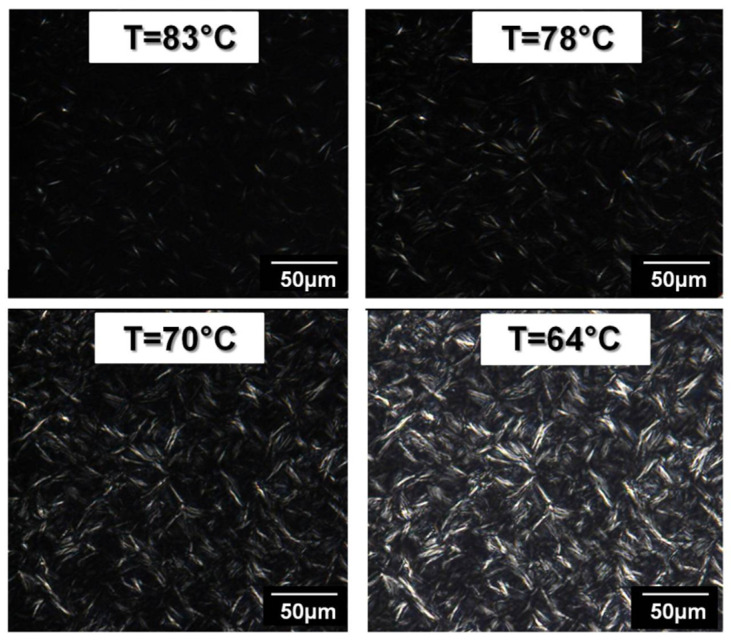
PE lamella crystallization in PE-b-PEG C (83 wt. % PEG) observed by optical microscopy in combination with polarized light during a cooling ramp.

During the cooling ramp, lamellas are growing between 83 °C and 64 °C as presented in Figure 12. These crystallization temperatures are close to the one observed for PE homopolymer. These lamellas could be then attributed to PE block crystallization. In PE homopolymer, the lamellas were oriented in a way to form spherulites. This modification of crystalline morphologies confirms the hindrance of the PEG blocks on PE block crystallization when PEG proportion is preponderant in the copolymer.

From the video recording, it was possible to determine the size and the growth rate evolution of the PE lamellas as a function of the crystallization temperature. Figure 12 shows that lamellas reach up to 11 µm in length. These PE lamellas in PE-b-PEG C are smaller in comparison with lamellas present in spherulites of PE homopolymer able to reach a length equal to 40 µm.

**Figure 13:**
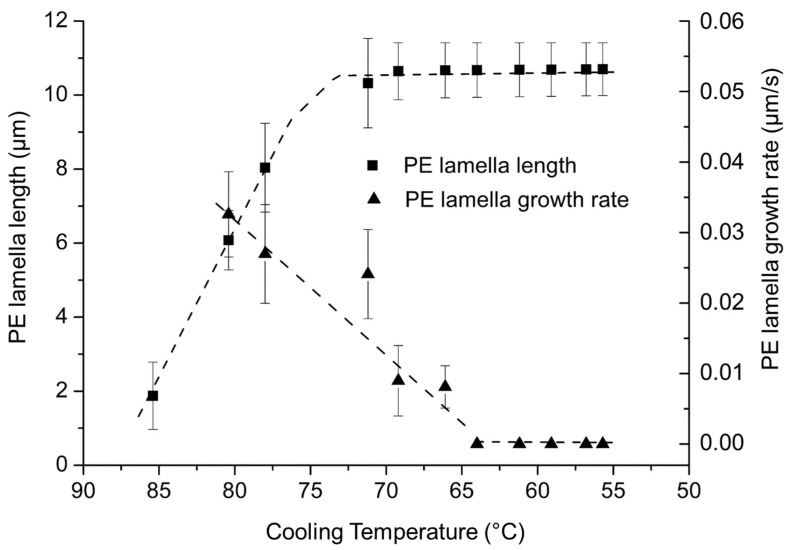
Evolution of the PE lamella length and growth rate as a function of the cooling temperature determined by optical microscopy for PE-b-PEG C.

The growth rate as a function of the crystallization temperature of the PE lamellas in the diblock PE-b-PEG C is presented in Figure 13.

To reach its final size, PE lamellas require a wider range of temperature in the case of the copolymer compared to the PE homopolymer. This might be explained by the fact that the PEG blocks are still melted at the crystallization temperatures of PE. The growth rate of PE lamellas is almost ten times slower than the lamellas growth rate of PE homopolymer. The growth rate of the PE blocks crystalline structures is significantly modified by the PEG block, probably due to its melted state hindering the lamellas growth.

**Figure 14:**
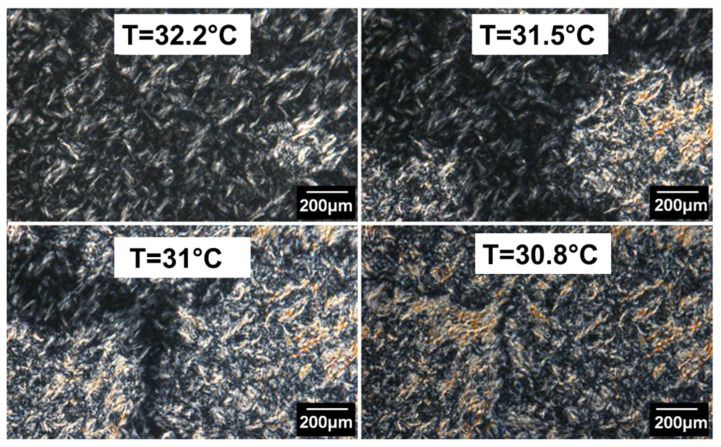
PEG spherulite crystallization in PE-b-PEG C observed by optical microscopy in combination with polarized light during a cooling ramp.

When the cooling temperature goes down to lower crystallization temperatures, crystallization of spherulites is observed around 32 °C through the PE lamellas already crystallized, as shown in Figure 14. These morphologies are attributed to the PEG block crystals because they appear at a temperature very close to the crystallization temperatures of pure PEG. These PEG block crystals completely cover the polymer layer. The diameter of the spherulites varies from 200 µm to 800 µm, similar to the spherulites of the PEG homopolymer.

**Figure 15:**
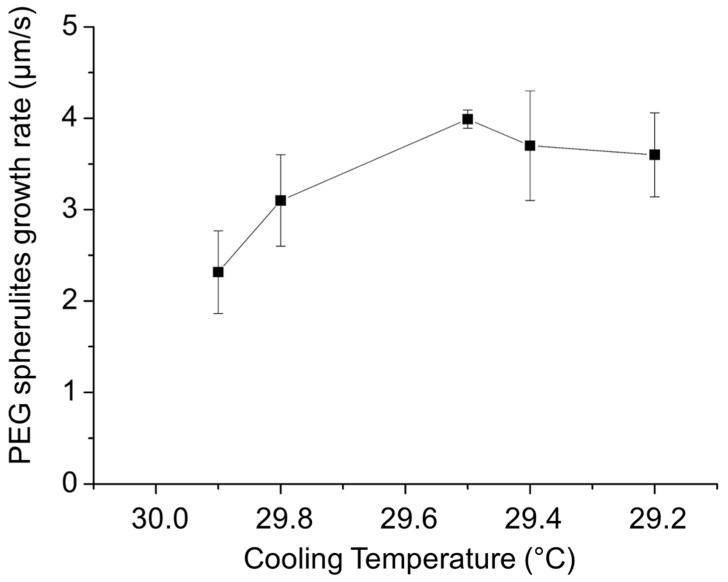
Evolution of PEG spherulite growth rate as a function of the cooling temperature detemined by optical microscopy in PE-b-PEG C (83 wt. % PEG).

The evolution of the growth rate of these spherulites as a function of the PE-b-PEG C crystallization temperature is presented in Figure 15. Unlike PEG homopolymer in the diblock, the PEG spherulite growth occurs in a very narrow temperature range. The growth rate of PEG crystalline structures in the PE-b-PEG C copolymer reaches a maximum equal to 4 µm/s. The growth rate of PEG spherulites is thus more than four times slower than in PEG homopolymer. This value indicates an important hindrance of the PE block on the PEG block ability to crystallize, especially due to the solid state of the PE lamellas already crystallized.

The melting and the crystallization temperatures of each block have been determined by optical microscopy coupled to a hot-stage and compared to the temperatures obtained by DSC in Table 4. The thermal transitions associated to PE block crystallization or melting were difficult to determine by DSC due to the little amount of PE analyzed, as this block is present in a low proportion. The combination of the hot-stage with the optical microscope revealed to be a fruitful technique in order to determine the thermal transitions for the PE block.

DSC curves of PE-b-PEG C (Figure 11c) showed no PE melting peak, but PE lamella crystallization has been observed between 94 °C and 50 °C by optical microscopy. T_m initial_ for PEG determined by optical microscopy (35 °C) is low compared to the one observed by DSC (52 °C). As the PEG crystalline structures are less organized due to the PE block influence, they melt at lower temperatures and are thus more difficult to detect by optical microscopy.

##### PE-b-PEG A (23 wt. % PEG) Copolymer

The morphology of PE-b-PEG A containing 77 wt. % PE was observed by optical microscopy under cross polarization light. In order to obtain information about the crystalline structures of a copolymer containing a high proportion of PE block, a heating and a cooling ramp have been applied successively to the copolymer while a camera records.

**Figure 16:**
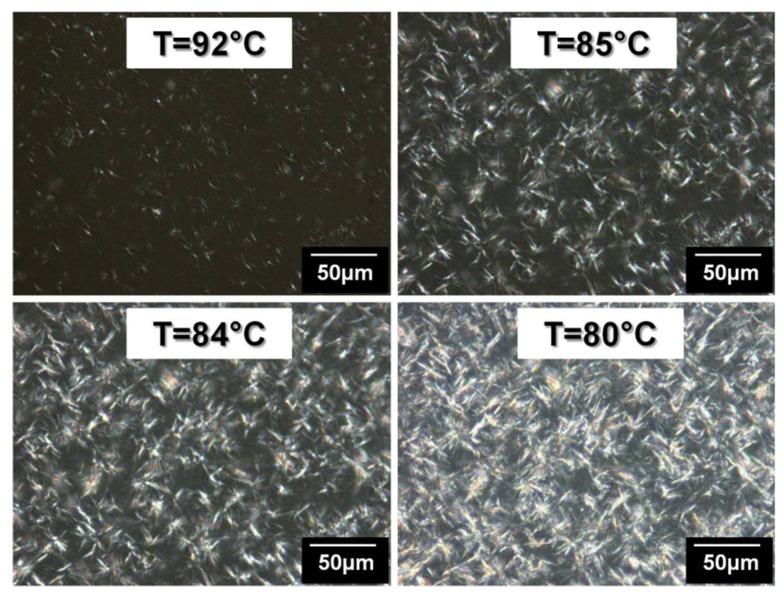
PE spherulite crystallization for PE-b-PEG A (23 wt. % PEG) observed by optical microscopy in combination with polarized light during a cooling ramp.

During the cooling ramp, PE spherulite growth is observed by optical microscopy from 95 °C to 70 °C. Some pictures of PE spherulite growth during the crystallization ramp of PE-b-PEG A are presented in Figure 16. The spherulitic-based crystallization is determined by the observation of a spherulite nucleus growing as the crystallization temperature decreases. It is interesting to point out that PE blocks crystallize as spherulites when PE sequences are dominating in the diblock. The PE blocks behave as pure PE in terms of the morphology of the crystalline structures.

**Figure 17:**
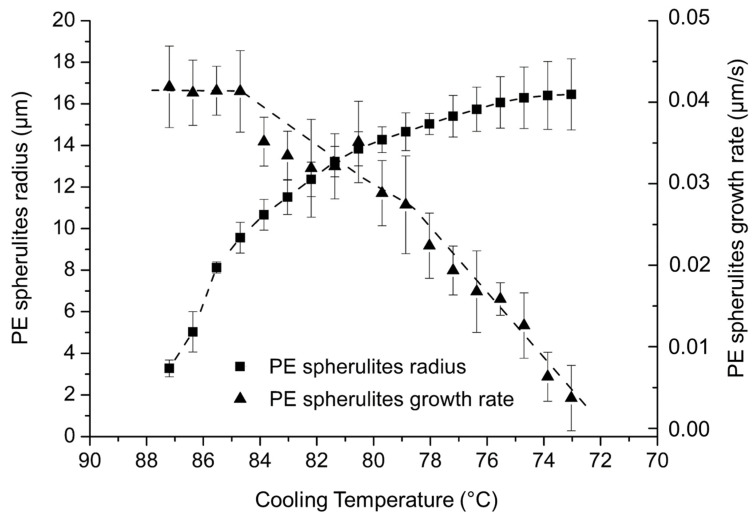
Evolution of the PE spherulite radius and growth rate as a function of the cooling temperature determined by optical microscopy for PE-b-PEG A.

Figure 17 presents the evolution of the size and the growth rate of PE spherulites as a function of the crystallization temperature in PE-b-PEG A determined from optical microscopy data. PE spherulite average radius reaches a maximum average size of 16 µm, which is small compared to the average radius of PE homopolymer spherulites of 40 µm. The PEG block, even in a reduced amount, tends to hinder the crystallization of the PE block.

The PE block crystallization temperature window is narrow for PE-b-PEG A relative to PE-b-PEG C due to the melted PEG blocks hindering the crystallization of the PE blocks in PE-b-PEG C. The growth rate decreases as the crystallization temperature of the diblock decreases, as observed for PE-b-PEG C. Figure 10 shows that PE homopolymer growth rate increases as the crystallization temperature decreases. The PEG block covalently linked to the PE block in PE-b-PEG reverses the trend of the PE growth rate as observed for the PE homopolymer.

No other crystallization event has been observed during the cooling of PE-b-PEG A. The PEG block crystallization was thus impossible to visualize by optical microscopy. As PEG blocks are in a reduced proportion (23 wt. % PEG), they either do not crystallize, or crystallize through the dense network developed by PE lamellas.

The melting and the crystallization temperatures of each block in PE-b-PEG A have been determined by optical microscopy and compared to the temperatures obtained by DSC in Table 5.

T_c final_ is observed at 70 °C by microscopy, but the crystallization process should go on until 1 °C according to the DSC data. The same gap is observed for the initial melting temperature, as the secondary crystallization is not as visible as the primary one is by optical microscopy.

##### PE-b-PEG B (55 wt. % PEG) Copolymer

In order to better understand the crystalline morphology of each block depending on the composition of the copolymer, PE-b-PEG B has been investigated by optical microscopy under polarized light. This copolymer has an intermediate composition relative to PE-b-PEG A and PE-b-PEG C. PE-b-PEG B presents a single melting peak and a single crystallization peak for each block.

**Figure 18:**
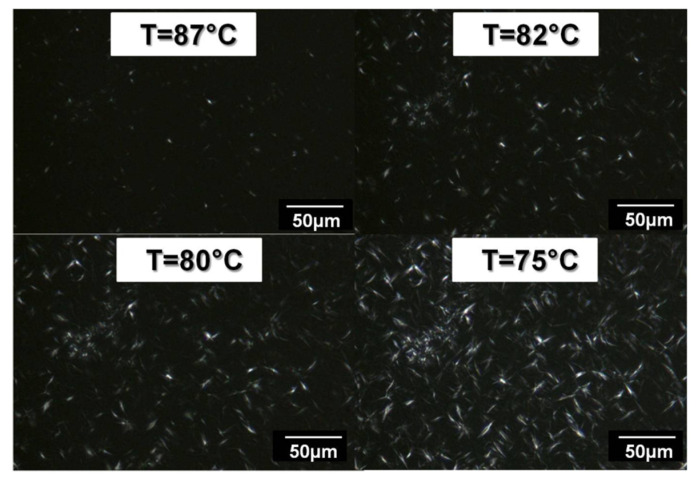
PE lamella crystallization for PE-b-PEG B (55 wt. % PEG) during a cooling ramp observed by optical microscopy in combination with polarized light.

A heating and a cooling ramp have been applied successively to the copolymer, while a camera records these processes. Figure 18 presents the first crystallization of PE-b-PEG B.

**Figure 19:**
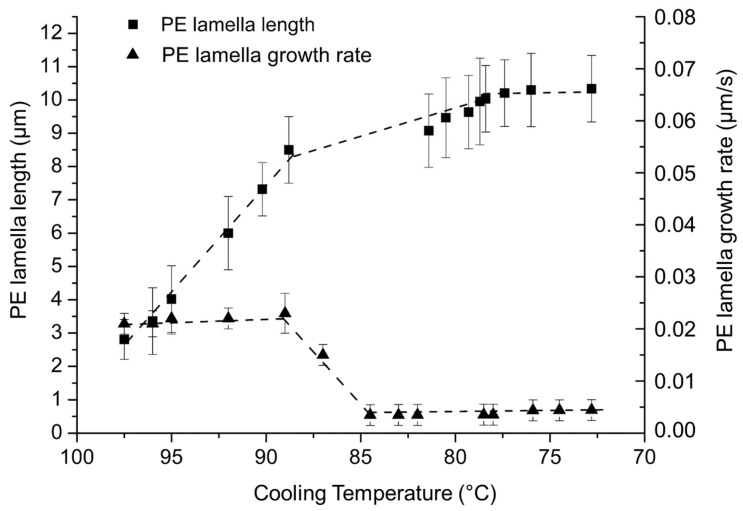
Evolution of the PE lamella length and growth rate as a function of the cooling temperature determined by optical microscopy in PE-b-PEG B.

During the cooling ramp, PE lamellas grow between 98 °C and 70 °C (see Figure 19). The PE block is in lower proportion in the copolymer (compared to the PEG blocks), but its content is sufficient to enable the PE blocks to crystallize in spherulites as they do in the PE homopolymer. The evolution of the size and the growth rate of PE lamellas as a function of the crystallization temperature are also presented in Figure 19.

PE lamellas reach an average length of 10 µm at their maximum in PE-b-PEG B, which is close to the average length of the PE lamellas in PE-b-PEG C containing a high proportion of PEG block. This means that over 50 wt. % of PEG, the PE blocks are not able to crystallize as spherulites, due to the PEG block hindrance. The growth rate of PE lamellas is very slow, lower than 0.02 µm/s compared to the growth rate of PE spherulites in PE-b-PEG A (0.04 µm/s) containing a high proportion of PE blocks. The mobility of PE lamellas is reduced due to the influence PEG blocks covalently linked to the PE blocks, and also due to the viscosity of the melted PEG blocks during the PE crystallization, revealing an important hindrance of the PEG blocks on the PE lamellas growth.

The melting and the crystallization temperatures of each block in PE-b-PEG B have been determined by optical microscopy coupled to a hot-stage and compared to the temperatures obtained by DSC in Table 6. PEG crystallization is not visible during the PE-b-PEG B cooling from 120 °C to the ambient temperature. This is consistent with the fact that DSC curves of PE-b-PEG B present a crystallization range between 3 °C and −12 °C, lower than room temperature.

## 4. Discussion

The effect of PE-b-PEG composition on the crystalline phase and the morphology of each block was observed by DSC and optical microscopy coupled to a hot-stage. The PEG homopolymer crystallinity was investigated and was compared with the PEG block crystallinity in PE-b-PEG diblock copolymer. The crystallinity degree of PEG homopolymer is equal to 95% for a molecular mass of *M_n_* = 2041 g·mol^−1^. The high crystallinity of PEG homopolymer was also observed for PEG with *M_n_* = 1000 g·mol^−1^ and *M_n_* = 2000 g·mol^−1^, presenting a crystallinity degree of 92.7% and 94% [16], respectively. Increasing PEG chain length leads to a higher crystallinity degree of PEG [14], as described by Li et al. for PEG, with *M_n_*= 20,000 g·mol^−1^ corresponding to a crystallinity degree of 99.2% [16]. These data are set as a reference in order to understand how the crystallinity is modified in PE-b-PEG diblock copolymers.

Table 7 summarizes the size and the growth rate of PE and PEG block crystalline structures (determined by optical microscopy) and the crystallinity degree (determined by DSC) for PE and PEG homopolymers and for PE-b-PEG of various compositions.

In PE-b-PEG C containing 83 wt. % of PEG, the PEG block (*M_n_* = 1474 g·mol^−1^) is very crystalline, with a crystallinity degree equal to 82%, as described in the literature [16]. Relative to the PEG homopolymer, the lower crystallinity degree of the PEG block in the copolymer can be explained firstly by the PEG chain length being shorter for PE-b-PEG C than for the PEG homopolymer, and also by the contribution of the PE block, leading to less organized crystalline structures of PEG as observed on the DSC curve in Figure 11c. In PE-b-PEG diblock copolymers, the PEG block crystallinity increases as PEG block length increases, while the PE block length remains constant. Longer PEG chains favor interactions between PEG blocks, and therefore also the ordered state of the PEG blocks. The PEG block crystallinity increases as the PEG proportion (and consequently the PEG block length) increases: the PEG block crystallinity degree is equal to 82% and 16% for PE-b-PEG containing 83 wt. % and 55 wt. % of PEG, respectively. For PE-b-PEG A with a high content of PE, the PEG blocks crystallinity cannot be determined because its melting peak is not detectable by DSC due to the very wide PE block melting peak contribution.

The studied PE-b-PEG copolymers present molecular weights varying from 575 g·mol^−1^ to 2250 g·mol^−1^, with PEG proportion (and PEG block length) increasing with the copolymer molecular weight. The PE homopolymer crystallinity was investigated and was also compared to the PE block crystallinity in the PE-b-PEG diblock copolymer, as presented in Table 7. The crystallinity degree of the PE homopolymer is equal to 44% for a molecular weight of *M_n_* = 1630 g·mol^−1^. The crystallinity degree of PE with *M_n_* = 1700 g·mol^−1^ has been determined to be 68% in the literature [30]. The difference observed can be explained by the integration limits varying from one study to another due to the not sharply defined peak ranging between 30 °C and 111 °C. Richards [31] and Burch [32] observed that relatively low molecular weight polyethylenes crystallized more easily than higher molecular weights, as polymer chains are not able to fold away for very low molecular weights. Indeed, the molecular weight of the PE block in PE-b-PEG A containing 77 wt. % of PE is equal to 334 g·mol^−1^ (see Table 1) with a high PE crystallinity degree of 94%. It is also important to notice that the PE melting peak might be influenced by the PEG crystalline sequence contribution in the very large PE melting peak (see Figure 11a), and consequently the crystallinity of the PE block might be influenced in PE-b-PEG A.

Table 7 clearly indicates the influence of the PEG block length on the crystallinity of the PE blocks in PE-b-PEG copolymers. In PE-b-PEG C, the crystallinity degree of the PE block, (in low proportion) was not determined because the PE melting peak was not observed on the DSC curve in the PE melting area, indicating that the PE blocks were poorly able to crystallize (see Figure 11c). The PE block crystallinity increases as the proportion of PE increases, relevant to the PEG block hindrance on the PE crystallization. Moreover, the PE crystallinity (for similar PE chain lengths) increases as the PEG chain length decreases, indicating that the PEG chain length highly influences the PE crystallinity. In PE-b-PEG copolymers, the ability of a block to crystallize is highly favored when this block is in high proportion in the diblock and is highly influenced by the presence of the other block.

The melting temperature of PEG is not significantly modified by the presence of the PE block in PE-b-PEG, as the PEG block length does not vary significantly, as is the case for PEG homopolymer and PE-b-PEG C. The melting temperature of PE in PE-b-PEG C does not appear on Table 7, as it was not observed on the DSC melting curve in Figure 11c.

For PE-b-PEG B, with a low proportion (i.e., shorter blocks) of PEG, T_m_ decreases due to the lower crystallinity degree of PEG and also due to the lower energy required to melt shorter PEG chains. It is also important to notice that the melting temperature of PE blocks in PE-b-PEG A is very low compared to the PE homopolymer. The shorter PE chains in PE-b-PEG A compared to PE homopolymer is able to explain this lower T_m_. A partial contribution of the PEG block melting, merged with the PE melting zone, is also able to induce this very wide peak, shifted to lower temperatures.

The PE crystallinity decreases when the PEG block ratio increases in the copolymers, and becomes negligible when the PE block ratio is equal to 17%, as in PE-b-PEG C. The PE block crystallinity decreases then as the content of PE decreases in copolymers, whereas the PE block length remains constant. A similar PE block length does not crystallize in the same way, as a function of the PEG block length. The longer the PEG block, the more numerous the PEG/PEG interactions (leading to a PEG crystallinity increase), and the PE/PE interactions are consequently reduced, leading to a decrease of the PE blocks crystallinity. The strong hindrance of the PEG block length on the PE crystallization is then evidenced by these results.

The previous explanations related to influence of PEG block length on PE crystallinity are also supported by the study of the morphology and the growth rate of the crystalline structures of each block, investigated by optical microscopy coupled to a hot-stage.

The PEG block crystallization was not observable for PE-b-PEG with a PE block proportion higher than 20 wt. %, such as PE-b-PEG A and PE-b-PEG B. On the other hand, the PE block crystallization was observed by optical microscopy for all PE-b-PEG copolymers. For high PEG content in the copolymer, as was the case for PE-b-PEG C, PEG blocks crystallize freely in large spherulites of average diameter equal to 200 µm as observed for the PEG homopolymer. It is important to notice that the growth rate of these spherulites is more than four times slower in the diblock containing 17 wt. % of PE than in the PEG homopolymer. The PE block induces then a hindrance on the PEG crystallization due to the PE crystalline phase already solidified at the PEG crystallization temperatures. Moreover, the PE lamellas are smaller in PE-b-PEG C than the PE spherulites observed for PE-b-PEG A, and growth is also slower.

When the PE ratio is majority in the copolymer, as is the case for PE-b-PEG A, PE blocks crystallize in very small spherulites of 16 µm radius, growing at 0.04 µm/s. It is important to notice that the growth rate of these spherulites is five times slower in the diblock containing 17 wt. % of PE than in the PE homopolymer. This rate is much slower than for the PE homopolymer due to the melted PEG sequences hindering the growth of PE crystalline structures. If the PEG ratio becomes more important, as is the case for PE-b-PEG B and PE-b-PEG C, PE is not even able to crystallize as spherulites anymore, but rather in lamellas.

The strong competition between PE and PEG block organization greatly influences the crystallinity of PE-b-PEG copolymers, as shown with the study of a copolymer with an intermediate composition. Indeed, PE-b-PEG B contains 55 wt. % of PEG, and the molecular weights of each block are quite equivalent. DSC curves of this copolymer (Figure 11b) showed that both blocks are able to crystallize, even if the PEG crystallization has not been clearly seen by optical microscopy. The PE blocks crystallize as spherulites, smaller than those observed for PE-b-PEG A, where the PE blocks are the majority, indicating that the PEG blocks hinder the growth of the PE spherulites in PE-b-PEG B. The PEG blocks are not able to crystallize as spherulites of 200 µm diameter, as is the case for the PEG homopolymer, because of the PE block hindrance leading to a lower PEG crystallinity degree in PE-b-PEG B.

## 5. Conclusions

Using DSC and optical microscopy coupled to a hot-stage, the bulk crystallinity of the PE-b-PEG diblock copolymers was investigated. According to the results presented in this paper, the effect of the blocks ratio and length was significant on the morphology and the growth rate of the crystalline structures. The crystallization of PE varies from spherulites to lamellas, depending on the proportion and the block length of PEG. When the ratio of one block is increased, the crystallization of that block is favored, while the crystallization of the other block is hindered, and could even be totally prevented.

The optical microscopy under cross polarization and coupled to a hot-stage appeared to be an appropriate and complementary tool to the DSC in order to get information about thermal transitions. These results, relative to the bulk crystallinity of PE-b-PEG diblock copolymers, could be compared, in a further study, to the organization of the same copolymers adsorbed, as a thin layer, on a solid substrate.

## Figures and Tables

**Table 1:** Molecular weight *M_n_* and chemical composition of studied polymers verified by proton nuclear magnetic resonance (^1^H NMR). Polyethylene-b-polyethylene glycol copolymers PE-b-PEG A, B and C are characterized by a PE block of constant length and a varying PEG block length.

	Chemical Composition Determined by ^1^H NMR					
Polymer	*M_n_* PE [g·mol^−1^]	*M_n_* PEG [g·mol^−1^]	PE Block [wt. %]	PEG Block [wt. %]	PE Block [molar %]	PEG Block [molar %]
PEG	-	2041	-	100	-	100
PE	1630	-	100	-	100	-
PE-b-PEG A	334	101	77	23	84	16
PE-b-PEG B	326	385	45	55	57	43
PE-b-PEG C	311	1474	17	83	25	75

**Table 2:** Comparison of melting and crystallization temperatures of PEG homopolymer determined by optical microscopy and DSC.

		DSC	Optical Microscopy
PEG crystallization temperatures (°C)	T_c initial_	39	40
	T_c final_	24	35
PEG melting temperatures (°C)	T_m initial_	43	49
	T_m final_	59	60

**Table 3:** Comparison of melting and crystallization temperatures of PE determined by optical microscopy and DSC.

		DSC	Optical Microscopy
PE crystallization Temperatures (°C)	T_c initial_	101	108
	T_c final_	19	50
PE melting temperatures (°C)	T_m initial_	29	30
	T_m final_	111	110

**Table 4:** Comparison of the melting and the crystallization temperatures of PE and PEG blocks for PE-b-PEG C obtained by optical microscopy and DSC.

PE-b-PEG C (83 wt. % PEG)		DSC	Optical Microscopy
PE crystallization Temperatures (°C)	T_c initial_	-	94
	T_c final_	-	50
PE melting temperatures (°C)	T_m initial_	-	76
	T_m final_	-	90
PEG crystallization temperatures (°C)	T_c initial_	37	33
	T_c final_	24	31
PEG melting Temperatures (°C)	T_m initial_	35	52
	T_m final_	58	58

**Table 5:** Comparison of melting and crystallization temperatures of PE and PEG blocks for PE-b-PEG A obtained by optical microscopy and DSC.

PE-b-PEG A (23 wt. % PEG)		DSC	Optical Microscopy
PE crystallization Temperatures (°C)	T_c initial_	90	95
	T_c final_	<10	70
PE melting Temperatures (°C)	T_m initial_	17	85
	T_m final_	96	98
PEG crystallization Temperatures (°C)	T_c initial_	-	-
	T_c final_	-	-
PEG melting Temperatures (°C)	T_m initial_	-	-
	T_m final_	-	-

**Table 6:** Comparison of the melting and the crystallization temperatures of PE and PEG blocks in PE-b-PEG B obtained by optical microscopy and DSC.

PE-b-PEG B (55 wt. % PEG)		DSC	Optical Microscopy
PE crystallization Temperatures (°C)	T_c initial_	87	94
	T_c final_	5	79
PE melting Temperatures (°C)	T_m initial_	47	60
	T_m final_	98	95
PEG crystallization Temperatures (°C)	T_c initial_	3	-
	T_c final_	−12	-
PEG melting temperatures (°C)	T_m initial_	28	26
	T_m final_	45	55

**Table 7:** Size and growth rate of PE and PEG crystalline structures determined by optical microscopy in combination with a hot-stage, and crystallinity degree (determined by DSC) for PE-b-PEG copolymers and PE and PEG homopolymers.

			PEG Crystalline Structures			PE Crystalline Structures		
Polymer	*M_n_* PEG Block (g·mol^−1^)	PE Block (wt. %)	Size (µm)	Growth Rate (µm/s)	Crystallinity Degree (%)	Size (µm)	Growth Rate (µm/s)	Crystallinity Degree (%)
PEG	2041	0	200	17	95	-	-	-
PE-b-PEG C	1474	17	200	4	82	11	0.03	0
PE-b-PEG B	385	45	-	-	16	10	0.02	27
PE-b-PEG A	101	77	-	-	0	16	0.04	94
PE	0	100	-	-	-	40	0.2	46
